# AI-Enabled, Ultrasound-Guided Handheld Robotic Device for Femoral Vascular Access

**DOI:** 10.3390/bios11120522

**Published:** 2021-12-18

**Authors:** Laura J. Brattain, Theodore T. Pierce, Lars A. Gjesteby, Matthew R. Johnson, Nancy D. DeLosa, Joshua S. Werblin, Jay F. Gupta, Arinc Ozturk, Xiaohong Wang, Qian Li, Brian A. Telfer, Anthony E. Samir

**Affiliations:** 1 Lincoln Laboratory, Massachusetts Institute of Technology, Lexington, MA 02421, USA; brattainl@ll.mit.edu (L.J.B.); lars.gjesteby@ll.mit.edu (L.A.G.); matt.johnson@ll.mit.edu (M.R.J.); nancyd@ll.mit.edu (N.D.D.); joshua.werblin@ll.mit.edu (J.S.W.); jay.gupta@ll.mit.edu (J.F.G.); 2 Department of Radiology, Massachusetts General Hospital, Boston, MA 02114, USA; ttpierce@partners.org (T.T.P.); aozturk@mgh.harvard.edu (A.O.); wangxiaohong426@hotmail.com (X.W.); li.qian@mgh.harvard.edu (Q.L.); asamir@mgh.harvard.edu (A.E.S.)

**Keywords:** vascular access, femoral vein, medical robotics, artificial intelligence, deep learning

## Abstract

Hemorrhage is a leading cause of trauma death, particularly in prehospital environments when evacuation is delayed. Obtaining central vascular access to a deep artery or vein is important for administration of emergency drugs and analgesics, and rapid replacement of blood volume, as well as invasive sensing and emerging life-saving interventions. However, central access is normally performed by highly experienced critical care physicians in a hospital setting. We developed a handheld AI-enabled interventional device, AI-GUIDE (Artificial Intelligence Guided Ultrasound Interventional Device), capable of directing users with no ultrasound or interventional expertise to catheterize a deep blood vessel, with an initial focus on the femoral vein. AI-GUIDE integrates with widely available commercial portable ultrasound systems and guides a user in ultrasound probe localization, venous puncture-point localization, and needle insertion. The system performs vascular puncture robotically and incorporates a preloaded guidewire to facilitate the Seldinger technique of catheter insertion. Results from tissue-mimicking phantom and porcine studies under normotensive and hypotensive conditions provide evidence of the technique’s robustness, with key performance metrics in a live porcine model including: a mean time to acquire femoral vein insertion point of 53 ± 36 s (5 users with varying experience, in 20 trials), a total time to insert catheter of 80 ± 30 s (1 user, in 6 trials), and a mean number of 1.1 (normotensive, 39 trials) and 1.3 (hypotensive, 55 trials) needle insertion attempts (1 user). These performance metrics in a porcine model are consistent with those for experienced medical providers performing central vascular access on humans in a hospital.

## 1. Introduction

Timely vascular access is critical to trauma management, permitting the administration of emergency drugs, analgesics, and blood volume replacement [1]. While peripheral venous catheterization plays a key role in fluid resuscitation, central vascular catheterization allows reliable and durable vascular access, enables large fluid volume resuscitation, and facilitates invasive endovascular therapies [1]. Early intervention opportunities at the point of injury, during medical evacuation, in an emergency room, or at military forward surgical facilities depend on timely vascular access. Timely central vascular access is likely to have the greatest impact in those cases where the evacuation time to a hospital is longest, including in under-served communities [2] and in military settings, where the evacuation time is thought to be likely to increase to a day or more in future large-scale conflicts [3]. However, central vascular access is normally achieved by highly experienced critical care physicians in hospital settings that are typically distant from the site of injury. Critical barriers to obtaining central vascular access in the field include a lack of in-field advanced ultrasound image interpretation skills and proficiency with image-guided vascular needle placement. Our work addresses these issues, enabling medical providers with minimal vascular access expertise to proficiently obtain central vascular access in prehospital settings.

Central vascular access is more challenging than peripheral venous access because blood vessels are deeper, precluding direct visualization and confounding manual palpation. Major arterial structures are typically in close proximity to the targeted veins, increasing the risk of inadvertent arterial injury. Despite the risk and complexity, over seven million central venous catheters are placed each year in the USA [4]. For fluid resuscitation, target vessels include the common femoral, internal jugular, and subclavian veins. Other interventions such as resuscitative endovascular balloon occlusion of the aorta (REBOA) [5] require femoral arterial cannulation. Extracorporeal life support requires access to both the femoral vein and artery, to the femoral and internal jugular veins, or to the internal jugular vein alone [6]. Ultrasound guidance offers critical advantages over landmark- and palpation-based approaches in that ultrasound mitigates problems caused by anatomic variability, vascular occlusion (such as from thrombosis), or absence of a palpable pulse, which can be caused by hypotension, cardiac arrest, or a ventricular assist device [7].

Despite the value of ultrasound-guided central access, the procedure is currently rarely implemented in prehospital settings because both ultrasound image interpretation and catheter insertion require extensive training and practice. Catheter insertion following the standard Seldinger technique [8] has multiple steps that include: (1) needle puncture of a target vessel, (2) coaxial guidewire insertion, (3) needle withdrawal, (4) iterative puncture-site dilation as needed, (5) over-the-wire catheter insertion, and (6) guidewire removal. The step that requires the most expertise, and is most prone to failure, is the initial needle insertion. Furthermore, successful completion of the guidewire insertion and needle withdrawal almost guarantees the success of the subsequent steps and eliminates the risk of needle injury.

To enable operators with varying amounts of medical training to obtain central vascular access, we prototyped AI-GUIDE (Artificial Intelligence Guided Ultrasound Interventional Device). Key contributions of this work include: (1) a handheld device that locates the femoral vein via AI-enabled ultrasound interpretation, performs targeted motorized needle insertion, automatically confirms successful vascular puncture, and assists with guide wire insertion; (2) phantom and porcine test results that show that AI-GUIDE enables expert and novice users to rapidly localize a vessel without the need for manual ultrasound image interpretation; (3) porcine test results that show that AI-GUIDE enables a user to execute the Seldinger technique with an accuracy and speed that is consistent with previously published results for experienced users, under both normotensive and hypotensive conditions. These results are based on an operator completing less than 10 min of training, following a simple (non-ultrasound) display interface to move the device to an optimal needle insertion location, and pushing a button to actuate the needle insertion. A key innovation in the AI-GUIDE system comprises complete end-to-end AI guidance of ultrasound-guided interventional needle insertion without any need for manual ultrasound imagery interpretation or advanced needle insertion skills.

## 2. Background and Related Work

This section includes background on femoral vascular anatomy and the standard method for performing femoral vascular access, and related work on robotic vascular access.

**Figure 1:**
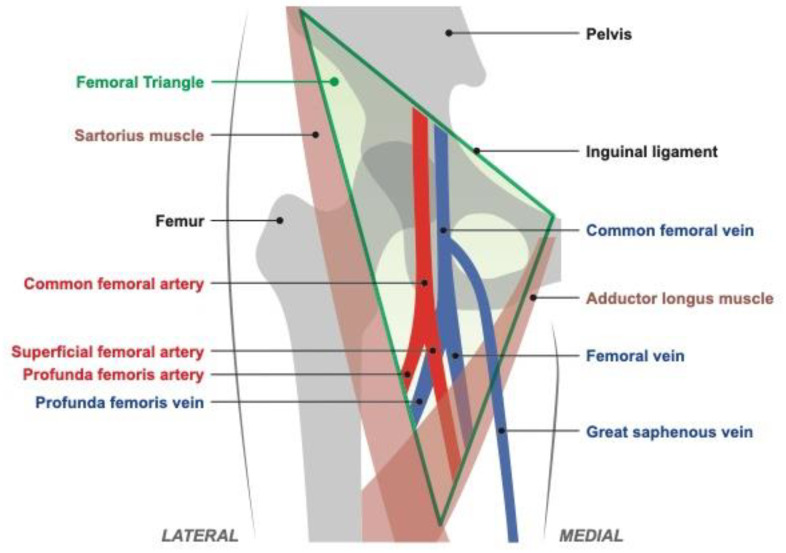
Femoral blood vessel anatomy [14] (art modeled after [15]).

The common femoral artery and vein are cannulated in the femoral triangle shown in Figure 1, with the anatomical parameters given in Table 1. The common femoral vessels are bounded cranially by the inguinal ligament and caudally by their bifurcation into deep femoral and superficial femoral distal branches. Cannulation above the inguinal ligament, which separates the abdominal cavity and the thigh, increases risk of uncontrollable retroperitoneal hemorrhage [9,10], while insertion below the artery bifurcation increases the risk of pseudoaneurysm and arteriovenous fistula formation [11,12]. When performed in the optimal region, the risk of pseudoaneurysms and arteriovenous fistula is low and bleeding complications can be easily managed with direct pressure. The length of that region is referred to as the “access length” in Table 1 and is, on average, 8 cm. Anatomic variability in the medial–lateral position of the neurovascular bundle within the femoral triangle and vessel depth both pose further challenges for needle targeting. Additionally, in 65% of people, the common femoral artery partially overlaps the common femoral vein in the anteroposterior plane [13], which places the artery at additional risk of injury during attempted venipuncture.

Even with optimal needle targeting, as the needle impinges on the vessel the walls of the vein may push inward or “tent”, preventing needle penetration. The stiffer arterial walls may slide medially or laterally, which may cause the needle to pass to one side of the artery, or to penetrate off-center, potentially resulting in an arterial tear or dissection. Under conditions of blood loss there is a decline in intravascular pressure and a compensatory decrease in vessel diameter, resulting in a smaller target and increased tenting.

Key performance metrics for vascular access include first-attempt success percentage, number of needle insertions, and time required for vessel cannulation. For experienced clinicians performing ultrasound-guided femoral artery access in a hospital, Seto et al. [19] found an 83% first-attempt success rate, a mean number of 1.3 attempts, and a mean time to sheath insertion of 185 ± 175 s. This study also reported a higher first-attempt success rate with a needle guide compared to without (84% vs. 70%) and a faster access time for experienced ultrasound operators compared to novice operators (mean of 158 s vs. 268 s). Similar results have been found for femoral vein access in a hospital, with a mean first-attempt success of 86% and a mean number of 1.16 attempts [20]. In a prehospital setting, both peripheral and central (subclavian and internal jugular veins) access attempts were found to require a mean number of 1.3 attempts and mean time of 2 min to access by emergency nurses and physicians [21,22]. Manley et al. [23] reported on three cases of combat casualties for whom a surgeon or emergency medicine physician performed ultrasound-aided femoral arterial access followed by REBOA in a prehospital setting. The time to balloon occlusion (including femoral access) was 5–7 min.

Progress has been made on assisting operators to perform peripheral access. A handheld robotic venipuncture device designed to perform blood draws on peripheral forearm veins has been tested on 31 human subjects with a 25G hypodermic needle [24]. This device integrates ultrasound imaging and two-degrees-of-freedom (DOF) robotic needle insertion at a fixed angle of 25 degrees. Ultrasound image interpretation and device positioning was performed by a physician, after securing the arm to a benchtop. A venipuncture success rate of 87% was achieved, with a mean number of 1.2 insertion attempts, and an average procedure time of 93 ± 30 s. The mean vessel diameter was 6 mm, and vessel depths of greater than 8 mm were excluded. Achieving clinically relevant femoral access in injury settings requires insertion of larger needles more deeply (up to 4 cm). For modestly trained operators to accomplish this goal in out-of-hospital settings requires multiple additional innovations, including autonomous vascular detection, localization, and classification, and development of a simplified interface to facilitate highly accurate needle targeting.

A larger, benchtop autonomous version of the peripheral venipuncture technology integrates infrared and ultrasound sensing and deep learning, for vein selection and needle insertion [25]. Results of image processing testing on humans and needle insertion testing on phantoms and submillimeter rat vessels were comparable to, or exceeded, clinical standards. However, the device does not specifically address the additional challenges of central vascular access and its large size, weight, and power make it unsuitable for prehospital applications or critically ill patients who cannot be easily transported to a fixed device. Indeed, Leipheimer et al. note that the technology’s “large size, lack of mobility, and large number of DOF” make it unsuitable for emergency use [24].

Other work has focused on automatically detecting blood vessels and nerves in ultrasound imagery to aid users in gaining vascular access and performing nerve blocks. Smistad et al. [26] reported results for detecting peripheral vessels in the forearm using neural networks. Precision and recall values of above 0.8 were achieved, but this operating point was considered too low for clinical utility. The detection of femoral vessels was also considered, with a resulting accuracy of 94.5% [27]. No attempt was made to discriminate between the femoral vein and artery. This work considered ultrasound image analysis only and did not integrate the research with human or robotic vascular access. In other work, the common carotid artery and internal jugular vein were tracked and perfectly classified in ultrasound videos from 38 subjects [28].

More broadly, precision robotic needle insertion has been an active area of research, as recently reviewed in [29,30]. Applications include a wide range of biopsies and cancer treatments such as radiofrequency ablation.

The emphasis of the research reported in this paper was on high accuracy and precision for both vessel detection and needle insertion, together with low size, weight and power and automation for use by operators with varying levels of skill, including non-experts.

## 3. Materials and Methods

An overview of AI-GUIDE is given in Section 3.1, with the AI and handheld robotic subsystems detailed in Section 3.1.1 and Section 3.1.2, respectively. Testing methods are detailed in Section 3.2.

### 3.1. AI-GUIDE Device

**Figure 2:**
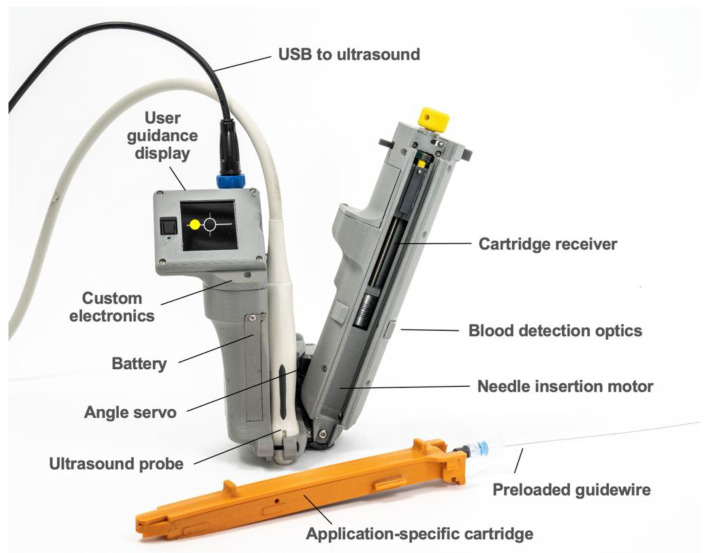
AI-GUIDE handheld robotic system.

AI-GUIDE consists of two major subsystems: (1) custom AI software to interpret ultrasound imagery, and (2) handheld robotics to insert a needle, confirm needle insertion, and assist the user in guidewire insertion. The handheld robotic subsystem (Figure 2) attaches to a linear high-frequency commercial ultrasound probe (Terason 15L4A, Burlington, MA, USA), and both the subsystem and probe connect to a commercial ultrasound tablet (Terason uSmart 3200T, Burlington, MA, USA) with a graphics processing unit (GPU) and integrated custom AI software. The AI software automatically detects and segments blood vessels on ultrasound images in real time (30 frames per second). Vessel location information is used by the AI software to guide the operator to move the device to an optimal needle insertion point. It is also used by the robotic subsystem to automatically adjust the needle insertion angle and calculate the needle insertion distance. When triggered by the user, the robotic subsystem deploys the needle with high precision and stops advancing the needle once the needle tip reaches the target location inside the vessel. Automated optical sensing of blood flashback confirms intravascular insertion, mimicking the confirmation process used by a clinician. All control electronics and embedded software are integrated into the device. A guidewire is preloaded for immediate advancement by the operator.

To operate AI-GUIDE, a user begins by placing the device in the inguinal region near the groin crease, moving the device from lateral to medial. When a target vessel enters the ultrasound field of view, the operator is automatically guided by the system to position the device via a simple dot-and-crosshairs display. Once properly positioned, AI-GUIDE directs the operator to deploy the needle and, when triggered, it deploys the needle to the AI-calculated target coordinates. Needle placement is automatically confirmed. If successful needle placement is not automatically confirmed, the operator may choose to direct the device to automatically withdraw the needle and try again until success is achieved. Following needle placement, a preloaded guidewire is then advanced by the operator coaxially through the needle–syringe apparatus, after which the needle is retracted, leaving the guidewire ready to facilitate the placement of diverse vascular cannulae.

**Figure 3:**
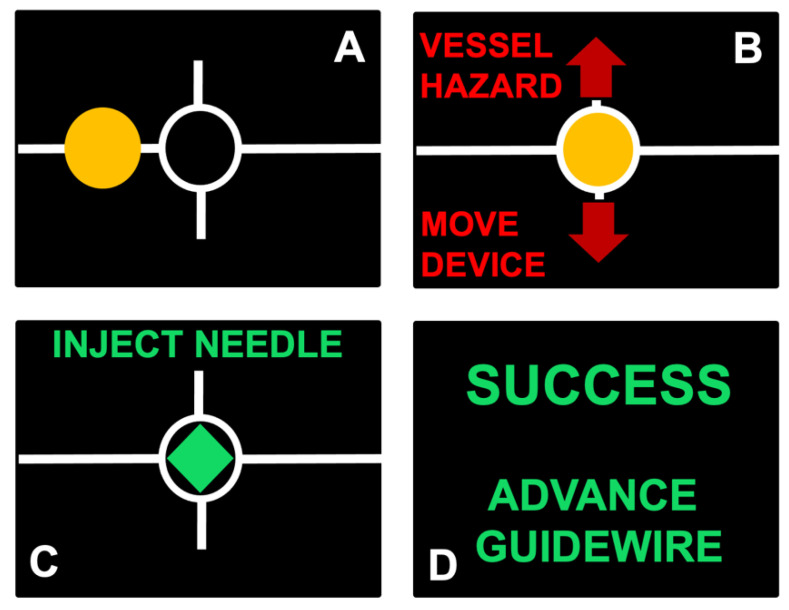
Selected views from user guidance display, (**A**) dot turns yellow as device approaches proper vessel position, (**B**) presence of hazards causes display to direct user to reposition device cranially or caudally, (**C**) dot turns green to indicate readiness for needle insertion, (**D**) automatic confirmation of needle insertion is displayed.

Images from the user guidance display shown in Figure 3 illustrate the guidance process. The lateral vessel location is translated into a simplified dot-and-crosshairs image on the display, allowing the user to intuitively position the device until the vessel is in the proper location for safe needle insertion. As the operator moves the probe, a red dot on the screen moves proportionally to the probe translation. When the operator approaches the proper vessel position, the dot turns yellow (Figure 3A) and the ratio of device movement to dot movement decreases in order to promote more precise positioning. Hazards, such as low positioning or overlapping vessels, will cause the display to direct the user to reposition the device cranially or caudally (Figure 3B). When properly positioned, the dot turns green to indicate readiness for needle insertion (Figure 3C). Needle insertion success is displayed once confirmed (Figure 3D).

#### 3.1.1. AI Software

**Figure 4:**
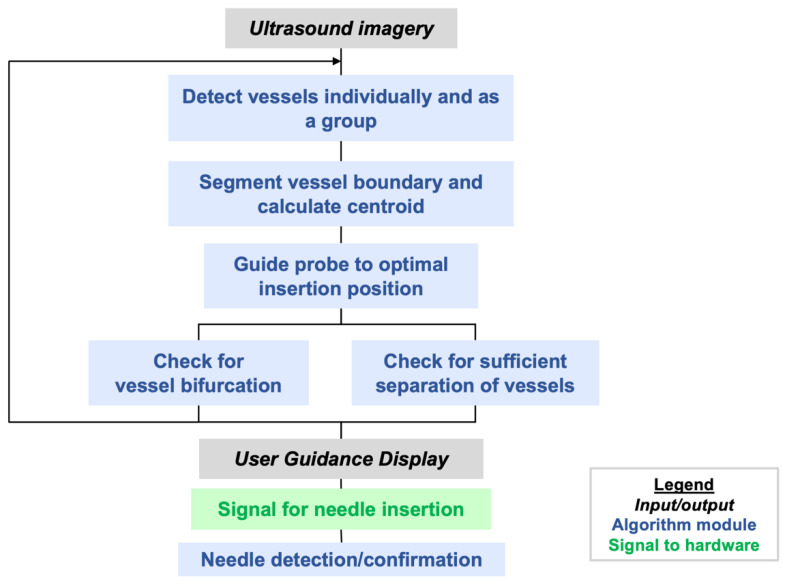
Flow diagram of informed AI for ultrasound-guided femoral vascular access.

The AI software is integrated into a multi-threaded C++ app that runs on a Terason uSmart 3200T ultrasound tablet in real time. The live ultrasound feed is the input to the AI application and the detected vessel locations and boundaries are the output to the handheld robotics subsystem. The purpose of the AI software is to emulate, in real time, the multiple thought processes of an expert clinician while inserting a needle into a deep vessel such as the femoral artery or vein. This is often termed “informed AI”, meaning AI informed in some way by human expertise or knowledge. Figure 4 illustrates the steps that are implemented in the AI software. Additional steps to be implemented in the future are mentioned in Section 5.

AI-GUIDE detects, classifies, and locates femoral vessels and bifurcations from transverse B-mode ultrasound views, with decision-making predicated on anatomic morphology alone. In clinical practice, additional sonographic techniques such as color Doppler, spectral Doppler, and dynamic compression may be used to confirm vessel patency [19,20]. These techniques are not included in the present AI approach as they add substantial additional technical complexity for relatively little yield, especially if applied in austere conditions with a low chance of occult vascular occlusion. Incorporation of these sonographic techniques would also require additional scanning time, which would delay vascular puncture. Lastly, Doppler and compression maneuvers may be unreliable in hypotensive patients due to slow flow, vasoconstriction, or venous collapse.

We adapted the You Only Look Once (YOLO) v3-Tiny network [31,32] to detect and compute bounding boxes on the artery, vein, and bifurcations. The initial YOLO weights were trained on the ImageNet database, which includes millions of natural images [33]. To adapt YOLO to ultrasound, we performed transfer learning [33,34,35,36] by retraining the weights using a curated database consisting of 19,000 annotated B-mode images of the porcine femoral region that were collected as part of the protocol described in Section 3.2. Transfer learning is one of the techniques we used to build high accuracy models without having to train the model on a very large ultrasound vessel dataset. As the ultrasound database was collected, we iteratively applied an active learning framework [34,37] to reduce annotation time and cost. This process started by automatically annotating a set of porcine images using the YOLO network trained on a small number of clinician-labeled images. Domain experts then reviewed the algorithm-generated annotations and corrected errors when needed. The refined annotations were used to retrain the AI model, which then classified a new dataset as it became available from a subsequent porcine study. After 7 iterations of datasets, the burden of manual labeling was reduced to less than 10% of each new dataset. To improve generalization performance, data augmentation techniques were applied during each training epoch to create image variations with random intensity and affine transforms.

If vessel bifurcation is detected, the user is cued to move the probe cranially. If the vessels are detected with no bifurcations, the vessel boundaries and centroids are segmented with a contour fitting algorithm adapted from [28]. Our algorithm follows the morphological operations and spoke fitting in [28] but adds Otsu thresholding [38] to generate an initial binary image.

Once vessel boundaries and centroids are computed, safety logic is applied to recognize whether there is an untargeted vessel interposed between the device and the target vessel, i.e., an unsafe configuration for needle deployment. When this occurs, it is typically the femoral artery that partially or fully blocks the path to the targeted vein. In this case, the user is guided to continue scanning cranially and/or caudally until a clear path to the vein can be achieved. Finally, before giving a go-ahead for the user to initiate needle insertion, the AI logic will check that the distance between the estimated needle insertion point and vessel centroid is less than 10% of the vessel diameter, i.e., within 1 mm for a 1 cm vessel diameter. This dynamically computed insertion window thus provides the more precise positioning accuracy that is needed for smaller or deformed vessels (e.g., vessels in hypotensive conditions).

When the user initiates needle insertion, the software attempts to confirm insertion using image change detection. This mimics clinical practice, in which operators attempt to visualize the needle tip within the vessel to increase confidence that accurate placement has been achieved. This image-based confirmation is secondary to the blood flashback detection detailed in Section 3.1.2.

#### 3.1.2. Handheld Robotics and User Guidance Display

The AI-GUIDE handheld robotic subsystem consists of a reusable handheld robotic platform and a disposable application-specific cartridge (Figure 2). The reusable robotic platform consists of: (1) a 3D-printed housing, which forms a mechanically registered mounting location for the ultrasound probe, (2) a user guidance display, (3) a servo motor for needle angle control, (4) a motor and leadscrew system for needle insertion, (5) a needle injection button, (6) a battery, (7) blood detection optics for machine and user feedback, and (8) a custom printed circuit board for system power, signal conditioning and distribution, and microcontroller (Teensy 4.0) interfacing. The ultrasound system communicates with the device through a USB (universal serial bus) cable, and the device directs the gross and fine movements of the user via the user guidance display. The handheld robotic subsystem weighs 0.64 kg, is 23 cm long (needle insertion arm), and has a battery that can power approximately 80 needle insertions.

The robotic subsystem receives the following messages from the AI subsystem: (a) the lateral offset of the target blood vessel relative to the center element of the ultrasound probe, (b) the depth of the target blood vessel centroid and distance to the vessel posterior wall, and (c) a go/no-go indication of whether the projected needle path can safely reach the intended vessel without passing through an unintended vessel (safety logic). The lateral offset is output to the display to allow the user to optimize positioning. The blood vessel depth and vessel size are used to calculate the needle insertion angle and distance. Safety logic testing is a prerequisite for engaging the needle drive motor, and potential hazards are displayed on the visual interface for the user.

**Figure 5:**
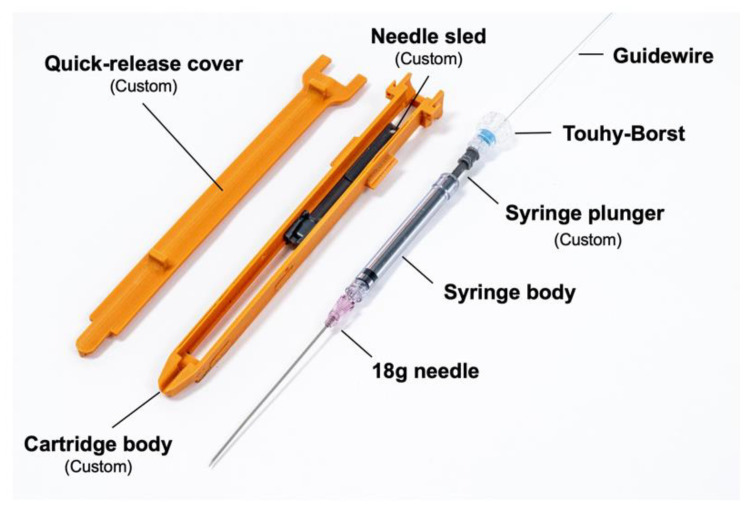
Cartridge and needle assembly for vascular access.

The disposable cartridge employed for the vascular access application, shown in Figure 5, consists of a 3D-printed body, a needle sled which interfaces the cartridge with a sliding element in the robotic platform, a 9 cm 18G angiographic needle (Merit Medical, South Jordan, UT, USA), an optically transparent 1 mL syringe body (Qosina, Ronkonkoma, NY, USA), a custom hollow syringe plunger, a Tuohy–Borst connector (Qosina), and a guidewire (Merit Medical MAK001, 0.018” × 40 cm, straight tip) preloaded via the Tuohy–Borst connector. A custom break-away door covers the needle elements while in operation and can be removed to eject the needle if desired.

The optically transparent syringe body, a light emitting diode (LED), and a photodiode comprise the blood flashback detection system. The LED (570 nm dominant wavelength) and photodiode are arranged such that the LED transmits light through the syringe body and onto the photodiode. When the needle advances into tissue, the syringe is extended, creating a vacuum. Blood is drawn into the syringe body once the needle tip penetrates the vessel wall. If air or water are in the syringe body, the photodiode detects light, but if blood is in the syringe, the blood blocks the light, indicating that the needle has been properly placed.

Needle insertion logic was developed to address two primary challenges during needle-insertion-device kickback and vessel tenting. Device kickback occurs as the needle pierces the skin at high speed and with force, generating a counterforce that could alter the device positioning and compromise insertion accuracy. The initial insertion speed (Table 2, waypoint A) was selected to minimize kickback while maintaining a short injection time, in order to prevent an operator from redirecting the device mid-injection. “Tenting” refers to the vessel wall being pushed inward by the impinging needle, impeding needle penetration. To address tenting, the needle is inserted past the estimated vessel centroid, but not passing through the posterior vessel wall, much as a clinician would do. This action of inserting past the centroid estimate is termed “overshoot”. If the blood flashback system does not detect blood, then the needle dithers back and forth to make another attempt at penetrating the tented vessel wall. These movements and the corresponding needle speeds are detailed in Table 2 as a series of insertion waypoints. The slower speeds during the dithering process were chosen to allow for the automated detection of blood flashback. The entire insertion process is accomplished within a maximum duration of about 3 s for a vein depth of 2 cm.

When blood is detected by the blood flashback system, needle motion is stopped, and the user is alerted on the display that the injection is successful, at which point the user can choose to advance the integrated guidewire and continue with the cannulation process. If no blood is detected at the end of the needle insertion process, then the user is notified and may press the needle injection button to retract the needle. The system will reset and allow immediate follow-on insertion to be initiated. It is possible to perform several needle insertion attempts within one minute, which improves the probability of successful needle insertion.

### 3.2. Test Methods

A series of tests were conducted to evaluate component and system function as well as usability by non-experts, as summarized in Table 3. The methods for each test are described in this section.

The AI subsystem was tested for vessel detection and classification accuracy, quantified as a precision–recall curve, and for vessel centroid estimation accuracy. Vessel bounding boxes and vessel centroids were annotated by experienced analysts. Performance was tested on a validation data set of 930 ultrasound images from a single pig that were held back from the training set. The independent test set consisted of the images that were classified during real-time system testing.

System tests were conducted on a phantom and porcine hemorrhage model to measure vessel acquisition time, vessel cannulation time, and needle insertion accuracies. In all tests, the users were presented with the simplified dot-and-crosshairs display and were blinded to the ultrasound display. Phantom testing was performed on the Gen II Femoral Vascular Access Training Model (Blue Phantom, Sarasota, FL, USA), constructed with materials that emulate the acoustic characteristics of human tissue (example ultrasound images available from [39]). As an initial test of vessel acquisition and needle insertion time for a range of experience levels, 11 users ranging in experience from a lab administrator (with no prior medical experience) to a senior radiologist with more than 30 years of experience were given a 2 min verbal tutorial on the device operation. Each user then operated the device on the femoral vascular access ultrasound training model three times, with the time to needle insertion and the success or failure recorded in each case. A board-certified radiologist with sub-specialty cardiovascular imaging training and 7 years of experience viewed the ultrasound display and the phantom “blood” flashback to assess success or failure.

Porcine studies were approved by the Massachusetts General Hospital (MGH) Institutional Animal Care and Use Committee (IACUC). Between October 2020 and February 2021, three female Yorkshire swine weighing 50–70 kg were housed overnight for acclimation under fasting conditions with free access to water. The animals were free of leptospirosis, mycoplasma hyopneumoniae, porcine reproductive and respiratory syndrome, swine influenza virus, brucella, toxoplasmosis, and pseudorabies. They were selected as the experimental model for all testing as they most closely match the human anatomy, tissue structure, and vascular structure size. On the day of the procedure, the animals were sedated, intubated, and maintained on 1–3% isoflurane for anesthesia. A surgically placed carotid artery catheter was used for invasive hemodynamic monitoring and exsanguination for initiation of hypotension. Upon completion of normotensive testing, hypotension was induced in each animal by controlled blood removal. Arterial pressure was maintained at 30 mmHg, as measured by a carotid arterial pressure monitoring catheter, by additional blood removal or intravenous fluid administration as needed. Upon completion of hypotensive testing, the animals were sacrificed via pentobarbital overdose.

To test vessel acquisition time in vivo, five users ranging from 0–15 years of medical training acquired the femoral vein on one sedated swine. The goal of the test was to center the target vessel in the image along the line of needle insertion in the shortest possible time. The operators used only directional information provided by the custom display interface and were blinded to the B-mode ultrasound imagery; they had no knowledge of vessel positioning beyond external surface landmarks. Once the user felt that the optimal location had been reached, an independent observer confirmed that the vessel was in the correct location for cannulation on the basis of ultrasound image review. The system was tested with and without the safety logic enabled (as described in Section 3.1.1).

Incremental device capabilities were tested by a single radiologist with 7 years of experience on the porcine hemorrhage model under both normotensive and hypotensive conditions. During testing, the operator maneuvered the device into position by viewing the user guidance display and was blinded to the ultrasound imaging. When in position, the operator deployed the needle and judged success on the basis of brisk blood return. Selected insertions were further confirmed by X-ray angiography. Repeated injections were performed on the bilateral femoral arteries and veins, as detailed in Table 3. A total of 39 needle insertion attempts were performed under normotensive conditions and 55 under hypotensive conditions. Complications such as arterial dissection and hematoma were recorded when observed sonographically.

Three porcine tests were conducted to quantify performance through controlled testing. The number of needle insertion attempts in these tests varied depending on the engineering goals of the test. For test 1, needle insertions were performed as independent trials. Tests 2 and 3 were performed with multiple attempts per trial, i.e., a first insertion would be attempted, and if the insertion was not confirmed automatically through blood flashback, the user would activate the device to withdraw the needle and a second attempt would be performed (or a third attempt if necessary). In a separate portion of porcine test 3, automated blood flashback detection was tested and the complete Seldinger technique was executed, including guidewire insertion and 4F catheter placement for confirmation.

Statistics are reported as means and standard deviations for comparison with previously published results, except for the results presented as box-and-whiskers plots, in which case statistics are reported as medians and interquartile ranges, to be consistent with the figures.

## 4. Results

### 4.1. Femoral Vessel Detection, Classification, and Localization

**Figure 6:**
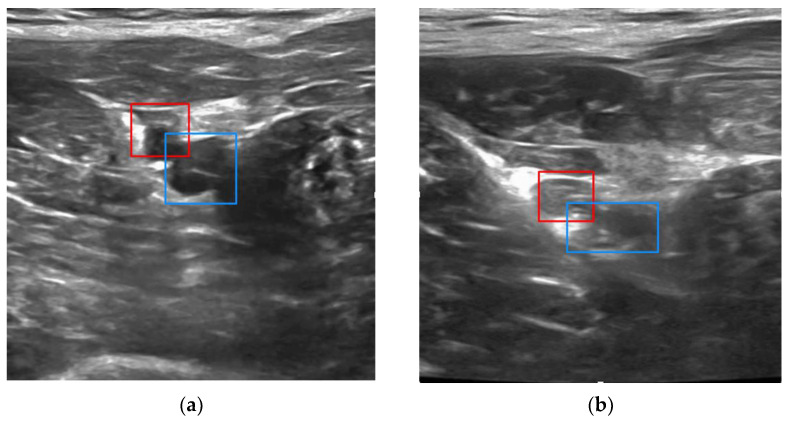
Examples of real-time vessel detection and classification, and bounding box placement in porcine study (femoral artery, red; femoral vein, blue) overlaid on ultrasound image: (**a**) normotensive condition and (**b**) hypotensive condition.

Examples of vessel detection and classification, and bounding box detection are shown for the right leg in Figure 6. (In the left leg, the artery would appear to the right of the vein in the ultrasound image.) To distinguish the artery from the vein, the YOLO network was trained to use morphological information, which could include the thicker and more hyperechoic arterial walls and differences in vessel cross sections, rather than a priori knowledge of which extremity is imaged.

**Figure 7:**
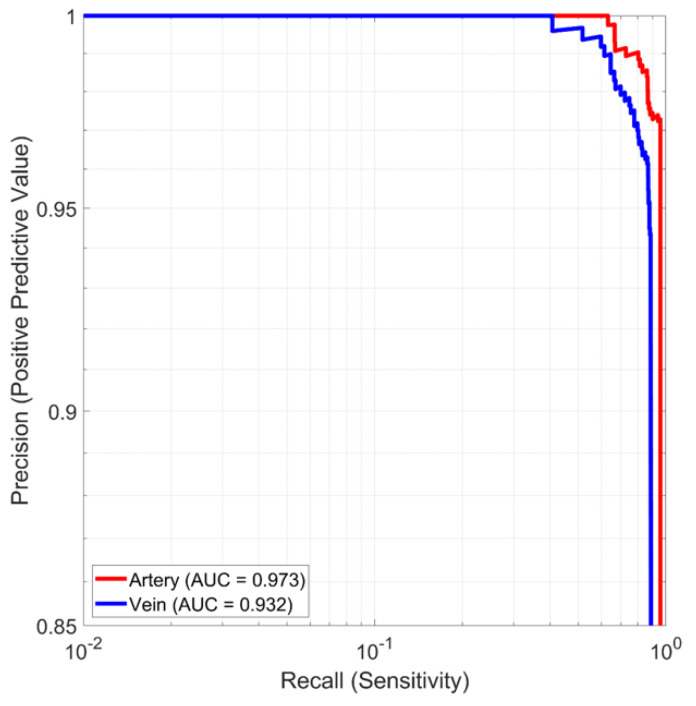
Vessel detection and classification accuracy.

Single-frame vessel detection was tested on 930 ultrasound images of the femoral artery and vein under both normotensive and hypotensive conditions, as used in the current AI-GUIDE system. Figure 7 plots the precision and recall for the detection of the artery and vein. The area under the curve (AUC) for the artery is larger than for the vein, at 0.97 (0.96–0.98) vs. 0.93 (0.92–0.95), which highlights the greater challenge of vein detection due to higher variability in morphology. For the operating points used in AI-GUIDE, the artery has 0.97 precision and 0.96 recall, while the vein has 0.94 precision and 0.89 recall. To mitigate errors based on these single-frame results, multi-frame logic is applied to smooth and improve input to the user display. In particular, if the current frame is missing a detection, detections from the previous frames are used. The results in Figure 7 cannot be directly compared to the 94.5% accuracy reported in [27] because the previous results detected blood vessels but did not attempt to classify the vessels as the femoral artery or vein or another vessel. To support needle insertion, the femoral artery and vein must be classified (i.e., distinguished), not simply detected.

**Figure 8:**
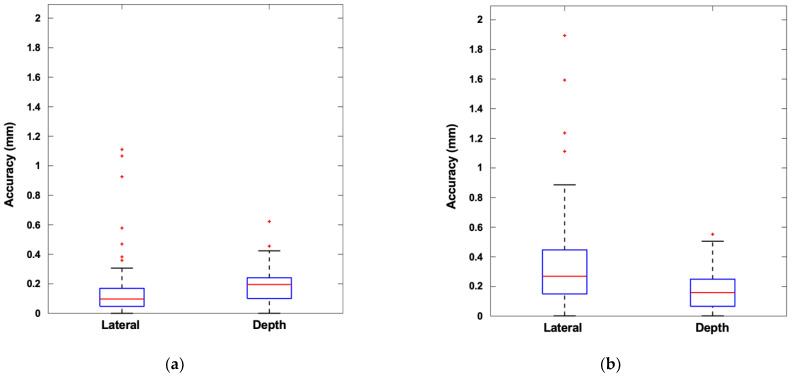
Vessel centroid estimation accuracy: (**a**) femoral artery; (**b**) femoral vein. Box-and-whiskers format displays median, interquartile range (IQR, box), ±1.5 IQR (whiskers), and individual data points outside of whiskers.

Vessel centroid estimation accuracy from the ellipse-fitting algorithm is quantified in Figure 8 for lateral and depth dimensions. The higher standard deviation of the vein localization accuracy once again results from the variability in vessel shape, size, and wall structure. Median accuracies are less than 0.3 mm in all cases.

### 4.2. Phantom and Porcine System Testing

**Figure 9:**
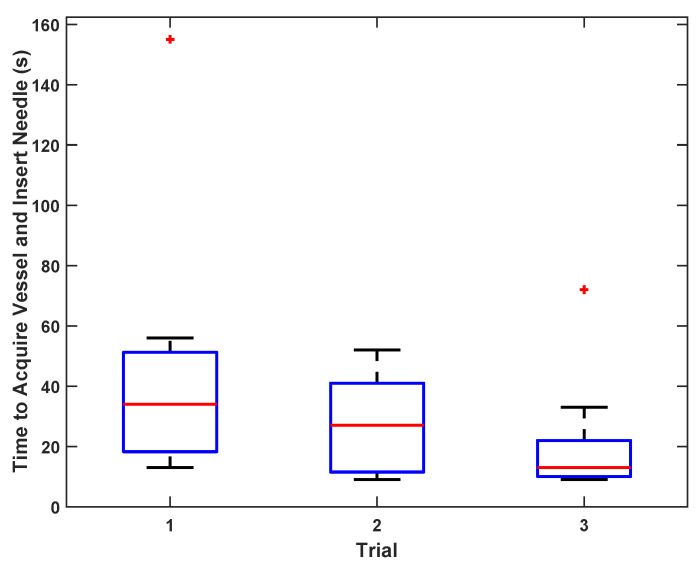
Times to acquire vessel and inject needle in phantom. Box-and-whiskers format displays median, interquartile range (IQR, box), ±1.5 IQR (whiskers), and individual data points outside of whiskers.

Vascular phantom testing showed that 11 users of varying experience successfully localized the target vessel and inserted a needle in less than 1 min (Figure 9). Median time to needle injection success decreased over the course of the three trials per user. By the third trial, median time to injection decreased to 13 (10–22) s. Safety logic was not applicable in phantom testing because the simulated artery and vein are side by side and have no antero-posterior overlap.

**Figure 10:**
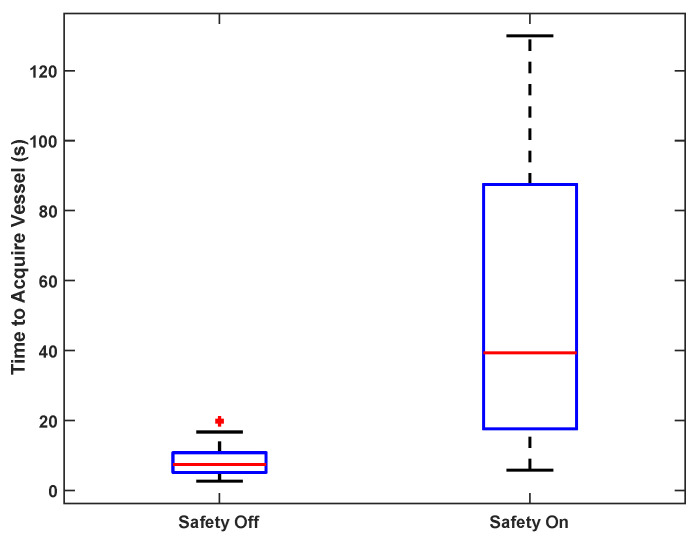
Times to acquire porcine vessel with safety logic off and on. Box-and-whiskers format displays median, interquartile range (IQR, box), ±1.5 IQR (whiskers), and individual data points outside of whiskers.

Vessel acquisition time testing for five users during porcine test 3 with safety logic on and off is summarized in Figure 10. With the safety logic disabled, the AI subsystem guided all five users to optimal insertion points within 20 s under both normotensive and hypotensive conditions. With the safety logic enabled, the median vessel acquisition time was 39 (18–88) s (mean 53 ± 36 s). This increase in acquisition time provides a sense of the additional complexity introduced by adding the important constraint of requiring a clear insertion path to the vein.

Table 4 provides the numbers of needle insertion attempts and successes during in vivo testing. The numbers of attempts varied across the tests depending on the engineering vs. success quantification objectives of each test. Overall, 92% (79–98%) of attempts were successful under normotensive conditions and 78% (65–88%) under hypotensive conditions. Considering each needle insertion attempt as independent, the mean number of attempts was 1.1 and 1.3, respectively. For test 3, the two-attempt success was quantified. In this test mode, if an initial needle insertion was not automatically confirmed, then the operator directed AI-GUIDE to fully withdraw and then re-insert the needle. For this scoring method, all 22 normotensive trials (21/22 first attempt success) and 21 of 22 hypotensive trials (17/22 first attempt success) were successful. The mean time from device contact on skin to needle insertion success was 48 ± 67 s. Retrospective analysis of unsuccessful attempts revealed vessel tenting to be the most common cause of failure.

As part of porcine test 3, automated blood flashback detection was tested in 12 trials (6 normotensive, 6 hypotensive). The needle was inserted in all cases in a single attempt, with blood flashback automatically detected in all cases. In the 6 normotensive trials, the complete Seldinger technique was executed, including guidewire insertion and catheter placement. The mean time to catheter placement was 80 ± 30 s.

The image-based needle insertion confirmation, which was investigated as a possible adjunct to blood-flashback confirmation, was also tested during a portion of porcine test 3. The algorithm ran in real time but was not used to provide feedback to the operator. The image-based confirmation successfully agreed with blood flashback presence or absence in 17 out of 17 normotensive trials and 17 out of 23 hypotensive trials.

## 5. Discussion

AI-GUIDE provides semi-automated needle and guidewire insertion with high needle insertion accuracy and speed that is comparable to that of experienced clinicians operating in hospital environments. The capability was demonstrated on a porcine model. The reported innovations comprise a critical departure from the previous automated vascular cannulation approaches that required a large nonmobile robotic arm. Instead, we combined innate human dexterity with machine learning and robotic precision to allow frontline providers, such as combat medics, emergency medical technicians, and paramedics to reliably obtain life-saving in-field central venous access.

During in vivo testing, AI-GUIDE achieved a high probability of needle insertion success comparable to that of experienced clinicians, requiring an average of 1.1 needle insertion attempts under normotension and 1.3 attempts during hypotension. Historically, experienced clinicians required a mean of 1.2 attempts to insert a needle in a human femoral vein [20] and 1.3 attempts to insert a needle in a femoral artery [19]. Our comparable results were achieved with the operator following only a simplified dot-and-crosshairs display with no knowledge of the underlying ultrasound imagery.

With respect to speed, experienced clinicians required a mean time of 185 s to insert a sheath in a femoral artery [19], compared to a mean time of 48 s for the AI-GUIDE needle insertion and 80 s for catheter insertion. For multiple users, including novices, the average time to needle insertion in a phantom was 21 s, indicative of the potential of AI-GUIDE technology to permit operators with minimal training and experience to successfully perform deep venous cannulation. This degree of success is likely to translate to in vivo situations, as shown by our smaller multi-operator cohort who were able to correctly localize the device in pigs in a mean time of just 53 s, which is well within a clinically acceptable time frame for femoral venous cannulation.

The purpose of this paper is to report on a proof of concept. Due to space limitations, additional experiments that evaluated trade-offs in design choices will be documented in a future publication. However, the following are key examples of improvements to the baseline system that were needed to achieve the reported results: (1) ellipse fitting to improve the vessel centroid estimate; (2) dynamic insertion window adjustment, which is particularly important for vessel size changes due to distortion or hypotensive conditions; (3) needle overshoot to overcome tenting; (4) needle insertion speed optimization to minimize device kickback when the needle strikes the skin surface; (5) improved ergonomics for device stability, including a two-handed grip, improved handgrip size, grip contouring to prevent slipping, and a handgrip aligned along the axis of needle insertion to counter kickback; (6) automated blood flashback detection to confirm successful needle insertion.

The potential impact of improved vascular cannulation spreads far beyond emergency care. Up to 80% of patients in intensive care units will require a central venous catheter with an associated immediate complication rate of 4–7% [4,40]. Operator training and standardized procedural checklists have been implemented to try to mitigate complication rates; however, utilization of AI-GUIDE may obviate errors from insufficient training and reduce complication rates further. Furthermore, AI-GUIDE may permit more cost-effective mid-level providers and skilled nurses to place central venous catheters, thus offloading work from busy physician intensivists. Additional venous interventions which may benefit from the accuracy and efficiency of AI-GUIDE include peripherally inserted central catheter (PICC) placement (2.7 million/year [41]), peripheral intravenous catheter placement (150 million/year [42]), and blood sampling via venipuncture (1.4 billion/year [24]). The millions of endovascular procedures performed each year, such as coronary angiography, peripheral arterial disease treatment, and aortic aneurysm repair, may also benefit from improved arterial access.

Beyond vascular access, there are a wide variety of applications that involve AI-enabled ultrasound-guided needle or catheter insertion. These include life-saving interventions such as a cricothyrotomy or tracheotomy for loss of airway and needle decompression for tension pneumothorax. Additional applications include abdominal fluid sampling and drainage, pleural fluid sampling and drainage, abscess drainage, tumor biopsy or ablation, targeted drug delivery of chemotherapy and novel therapeutics [43,44], cross-modality image fusion targeting, ultrasound-guided minimally invasive surgery, and even in-home patient-operated intervention. The modular and interchangeable cartridge design will simplify extensions to these planned additional applications.

There are a number of possible risks with the operation of this device that are similar to the risks of manual cannulation. Off-target needle insertion may cause damage to non-target structures, e.g., arterial injury when trying to cannulate the femoral vein. For applications elsewhere in the body, non-target injury is modulated by the proximity of adjacent critical structures. Vascular access in the upper arm would be considered low risk given the paucity of critical structures within a reasonable distance from the target, while internal jugular access would present a higher risk given the nearby carotid artery, trachea, and lung apex. Off-target injury is potentiated by algorithm false positive target identification (rare in this study), mechanical needle insertion error (minimal in this study), and operator movement during needle insertion (non-trivial). Iterative optimization of the form factor and needle insertion dynamics were critical for minimizing operator movement during needle insertion, yielding our high success rate. An additional risk is the delay in achieving vascular access and the resultant delay in definitive care. The current evidence suggests that AI-GUIDE may facilitate vascular access, especially if deployed in the field, although more study, including human trials, is warranted. Further risks, such as vascular injury/dissection, hemorrhage/hematoma, and thrombosis are likely to be comparable to standard of care manual vascular access, although further proof is needed.

This work has a number of limitations. The phantom had a fixed vessel configuration that may overestimate AI subsystem performance and allow users to fine-tune their acquisition approach during the course of three trials. To address this, we are developing a set of custom vascular phantoms based on CT (computed tomography) imaging of several human subjects. This is expected to improve the fidelity of phantom testing, allow more rapid device iteration, and reduce reliance on porcine testing.

There are several limitations that relate to the porcine testing. Firstly, possible complications could not be comprehensively assessed by gold-standard testing because it was not practical to perform standard of care CT angiography following each needle insertion attempt. However, our strategy of sonographic interrogation for complications mimics clinical practice, where routine CT angiography is not performed following each vascular cannulation attempt. One small femoral region hematoma was observed on ultrasound imagery in test 3, with safety logic applied. Any subsequent hematomas would be difficult to detect once extraluminal blood was present. Reassuringly, no large hematomas were identified. The complication rate for manual, ultrasound-guided femoral access performed in a hospital setting has been reported as 5.5% [20].

Secondly, testing on only three pigs (six legs) resulted in relatively large bounds on the accuracy estimates, even with multiple trials per vein. Utilization of only three animals may underestimate biologic variability and overestimate device performance. However, AI subsystem performance may be confounded by post-procedural changes following preceding attempts leading to underestimation of the true performance in future clinical practice. Nevertheless, the lower bounds of the 95% confidence intervals for needle insertion success (65% hypotensive, 79% normotensive) are sufficient to establish proof of concept, and the confidence bounds will improve with planned additional testing. The image processing and phantom testing provide additional support for the porcine results.

Thirdly, the pigs were similar in size and thus femoral vein depths were all approximately 1.5–2 cm, which did not allow for testing at the greater depths of up to 4 cm observed in humans [16]. We expect that operating over a greater range of depths will require an adaptive depth-of-view selection. (The depth of view used for the reported tests was 4 cm.) Other differences between porcine anatomy and human anatomy may confound our comparisons with prior human trials; however, pigs are a well-validated model of human cardiovascular research, and the differences for our application are anticipated to be small.

Fourthly, all tests were conducted in a quiet laboratory setting, on inanimate phantoms or anesthetized animals. Patient motion in the field due to pain or to vehicle (ambulance or helicopter) vibrations may complicate vessel cannulation efforts. These challenges may require additional innovations such as stabilization straps or the development of additional algorithms to mitigate motion effects. It is also possible that rapid mechanized needle insertions may mitigate the effect of patient motion, compared with relatively slow manual needle insertions. Similarly, the encapsulation of the needle associated with AI-GUIDE may increase safety for both the patient and the operator in unpredictable environments. These limitations will be addressed in future studies.

In addition to the documented success, we also highlight several ways in which our platform can be easily adapted to alternative hardware or use cases. Although our real-time AI software has been integrated into the Terason tablet, which supports the development of third-party applications with a software development toolkit, AI-GUIDE is designed to be easily translated to other portable ultrasound systems, such as those that consist of a smartphone connected to an ultrasound probe. For these systems, the integrated user guidance display in Figure 2 can be easily replaced by the smartphone display.

The amount of ultrasound information provided to the user can also be adapted to the level of user expertise. For an expert, the entire ultrasound image can be displayed, with vessel annotation overlays marking the desired vessel and target location. On the other hand, for a non-expert operating in a chaotic, distracting environment, the display can distill the key information from the ultrasound image that is required to center the device over the desired vessel.

In the reported results, testing on the curated ultrasound database and on phantoms is relevant to the femoral artery but full system testing on the porcine hemorrhage model was not conducted for the artery. In the future, full system testing will be performed for the femoral artery. In addition, the AI subsystem will be further improved and extended. One upgrade is to more accurately ensure that the needle is inserted in the vessel segments between the inguinal ligament and deep vessel bifurcation. The algorithm is also being extended to operate on the internal jugular vein, with peripheral veins also representing possible extensions. As AI-GUIDE progresses toward human testing, device considerations such as sterility are being addressed. There is also a desire to further reduce the size, weight, and power of the device, and further automate the guidewire and sheath placement.

## 6. Conclusions

A handheld AI-enabled interventional device, AI-GUIDE (Artificial Intelligence Guided Ultrasound Interventional Device) has been developed to direct users with no ultrasound or interventional expertise to catheterize a deep blood vessel, with an initial focus on the femoral vein. This capability is needed to allow emergency medical providers to save lives by performing central vascular access in military and civilian prehospital settings when a hemorrhaging patient cannot be rapidly evacuated to a hospital. Previous research on inserting a needle in a shallow peripheral vein using a large robotic arm does not meet this need. To achieve central vascular access, AI-GUIDE automatically detects and classifies the femoral artery and vein, identifies a safe needle insertion point, inserts a needle, automatically confirms proper insertion, and enables a user to insert a preloaded guidewire, followed by insertion of a sheath or catheter. The AI and robotics subsystems and AI-GUIDE system have been tested incrementally by users with a range of expertise using a combination of: (1) an ultrasound video database of the femoral region from humans and pigs, (2) synthetic femoral phantoms, and (3) a porcine model under both normotensive and hypotensive conditions. Initial test results indicate that AI-GUIDE can enable non-experts to perform femoral vascular access with a speed and success rate comparable to experts. Moreover, results indicate that AI-GUIDE can be successful in hypotensive conditions resulting from blood loss. The next steps are to mature the device and test it on humans under an FDA investigational device exemption.

## 7. Patents

Patent pending: Brattain, L.J.; Samir, A.E.; Telfer, B.A. et al. Systems and methods for portable ultrasound guided cannulation. Filed 17 August 2020, US20210045711A1.

## Figures and Tables

**Table 1:** Anatomical parameters relevant to human femoral vascular access. Depth is defined as distance from the skin surface to the anterior vessel wall.

	Common Femoral Vein	Common Femoral Artery
Depth [16]	2.2 ± 0.6 cm Range 0.9–3.8 cm	1.8 ± 0.6 cm
Diameter [16]	0.9 ± 0.3 cm	0.8 ± 0.2 cm
Access length	Mean 8 cm [17] Range 5–11 cm	Mean 7.5 cm [18]

**Table 2:** Needle insertion waypoints and insertion speeds.

Waypoint	Estimated Location	Mean Needle Insertion Speed to Waypoint (mm/s)
A: Initial overshoot	1 mm short of posterior vessel wall	45
B: Vessel centroid	Vessel centroid	20
C: Secondary overshoot	Posterior vessel wall	10
D: Semi-retracted position	Anterior vessel wall	10
E: Vessel centroid	Vessel centroid	10

**Table 3:** AI-GUIDE test summary.

Function Tested					Tested on	# Users	# Tests
Detect and Classify Vessels	Acquire Vessel	Insert Needle	Automatic Confirm	Place Guidewire and Catheter			
X					Curated Database	N/A	N/A
X	X	X			Phantom	11	33
X	X				1 Pig	5	20
X	X	X			3 Pigs	1	94
X	X	X	X		1 Pig	1	12
X	X	X	X	X	1 Pig	1	6

**Table 4:** AI-GUIDE needle insertion success.

Porcine Test	Needle Insertion Results (# Successful/# Attempted)	
	Normotensive	Hypotensive
1	6/8	8/10
2	8/8	14/16
3	22/23	21/29
Total	36/39 = 92% (79–98%)	43/55 = 78% (65–88%)

## Data Availability

The data presented in this study can be made available upon request pending U.S. Department of Defense approval.
